# Undoped Sr_2_MMoO_6_ Double Perovskite Molybdates (M = Ni, Mg, Fe) as Promising Anode Materials for Solid Oxide Fuel Cells

**DOI:** 10.3390/ma14071715

**Published:** 2021-03-31

**Authors:** Lubov Skutina, Elena Filonova, Dmitry Medvedev, Antoine Maignan

**Affiliations:** 1 Laboratory of Electrochemical Devices Based on Solid Oxide Proton Electrolytes, Institute of High Temperature Electrochemistry, 620137 Yekaterinburg, Russia; lubov.skutina@yandex.ru; 2 Institute of Natural Sciences and Mathematics, Ural Federal University, 620002 Yekaterinburg, Russia; elena.filonova@urfu.ru (E.F.); antoine.maignan@ensicaen.fr (A.M.); 3 Institute of Chemical Engineering, Ural Federal University, 620002 Yekaterinburg, Russia; 4 Laboratoire Crismat, UMR 6508 Normandie Université, CNRS, Ensicaen, Unicaen, 6 bd du Maréchal Juin, CEDEX 4, 14050 Caen, France

**Keywords:** SOFC, double perovskite, molybdates, anode materials, redox stability, tolerance, electrochemistry, LSGM, carbon deposition, sulfur poisoning

## Abstract

The chemical design of new functional materials for solid oxide fuel cells (SOFCs) is of great interest as a means for overcoming the disadvantages of traditional materials. Redox stability, carbon deposition and sulfur poisoning of the anodes are positioned as the main processes that result in the degradation of SOFC performance. In this regard, double perovskite molybdates are possible alternatives to conventional Ni-based cermets. The present review provides the fundamental properties of four members: Sr_2_NiMoO_6-δ_, Sr_2_MgMoO_6-δ_, Sr_2_FeMoO_6-δ_ and Sr_2_Fe_1.5_Mo_0.5_O_6-δ_. These properties vary greatly depending on the type and concentration of the 3d-element occupying the B-position of A_2_BB’O_6_. The main emphasis is devoted to: (i) the synthesis features of undoped double molybdates, (ii) their electrical conductivity and thermal behaviors in both oxidizing and reducing atmospheres, as well as (iii) their chemical compatibility with respect to other functional SOFC materials and components of gas atmospheres. The information provided can serve as the basis for the design of efficient fuel electrodes prepared from complex oxides with layered structures.

## 1. Introduction

The development of solid oxide fuel cells (SOFCs) capable of generating electricity by means of a single-step electrochemical approach has been an urgent task since the mid-nineteenth century. Currently, considerable efforts have been devoted to the study of both individual materials (electrolytes, anodes, cathodes, collectors, sealants) and the design and testing of SOFCs [1,2,3]. It is now extremely important to scale-up from laboratory activity to commercial device. Although many promising fundamental, theoretical and applied problems have been solved, others constantly arise, initiating the development of new technological approaches. For example, with the development of conventional SOFCs, in which YSZ (yttria-stabilized zirconia) acts as an electrolyte and Ni-based cermets serve as anodes, a cheaper and more accessible hydrocarbon fuel (natural gas, biogas, etc.) cannot be used. This is due to the degradation of nickel-based anodes caused by carbonization and sulfur poisoning, which leads to a decrease in SOFC lifetime [4,5,6,7,8,9]. The solution to this problem involves either modifying the Ni–YSZ composites by introducing various additives (thus preventing carbon deposition and improving chemical stability [10,11]) or searching for new anode materials that do not exhibit these disadvantages [12,13,14,15,16,17].

In terms of chemical design, there are many alternative materials for Ni-based composites. They include Mo-containing double perovskites with the general formula Sr_2_MMoO_6-δ_ [18,19,20,21,22,23,24,25,26,27,28,29,30]. The use of these compounds as anodes, in combination with La_1-x_Sr_x_Ga_1-y_Mg_y_O_3-δ_ (LSGM) electrolytes, allows for the operating temperatures of SOFCs to be lowered down to 800 °C (or even lower). This is due to the high electrical conductivity of LSGM electrolytes [31,32,33]. This aspect, along with the possibility of using Sr_2_MMoO_6-δ_ in hydrocarbon fuels, [34,35,36,37,38,39,40,41,42,43,44,45,46,47] opens up new directions for designing carbon- and sulfur-tolerant SOFCs.

The present review seeks to highlight and systemize information on the functional properties of Sr_2_MMoO_6-δ_ double perovskite molybdates (where M = Ni, Mg, Fe) in terms of their applicability to SOFCs. Special attention is devoted to analyzing their functional properties in both oxidizing and reducing conditions, both of which are used in SOFC fabrication and testing. On the basis of this analysis, the main advantages and problems are identified and future solutions proposed.

## 2. Brief Descriptions of SOFCs

**Figure 1:**
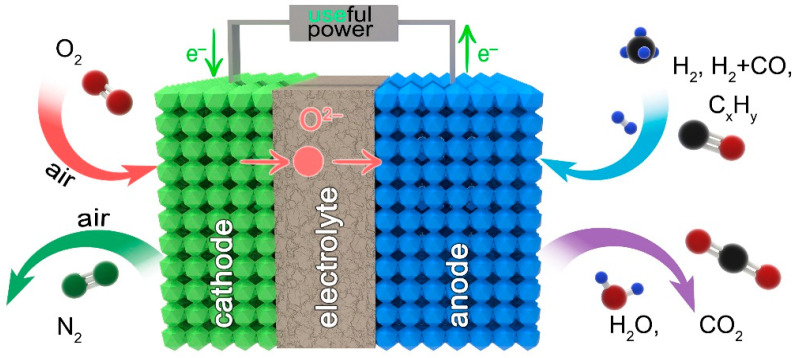
Simplified scheme of solid oxide fuel cells (SOFCs) working principle.

The extensive body of research on SOFCs is mostly motivated by the fact that they can efficiently generate electricity over a wide temperature range, do not contain liquid phases, do not require the presence of noble metals and allow for the use of various types of fuels [48,49,50,51,52,53,54,55,56]. An SOFC unit is a multilayered structure consisting of an ionic conductor (electrolyte) between two electrodes, an anode and a cathode (Figure 1).

During the operation of an SOFC, the cathode produces an oxygen reduction reaction (Reaction (1)) and delivers O^2−^ ions to the contact point with the electrolyte.

(1) $$O_{2}{+4e}^{-}{\;=\;2O}^{2-}$$

The function of the gas-tight electrolyte is to electrochemically transport the formed oxygen ions from the cathode to the anode, where they oxidize the fuel (Reactions (2)–(4)) and form generate electrons; the latter go through an external circuit to the cathode, thus forming potentially a power source. It should be noted that the electrolyte must block the flow of electrons from the anode to the cathode inside the cell, thus exhibiting unipolar (ionic) conductivity.

(2) $$H_{2}{+\;O}^{2}{}^{-}{=\;H}_{2}O+2e^{-}$$

(3) $${\mathrm{CO}+\;O}^{2}{}^{-}=\;CO_{2}+2e^{-}$$

(4) $${\mathrm{CH}}_{4}{+\;4O}^{2}{}^{-}=\;CO_{2}+2H_{2}O+8e^{-}$$

The overall reactions occurring in SOFCs when hydrogen, a synthesis gas or methane are used can be expressed by Equations (5)–(7), respectively.

(5) $${2H}_{2}{+\;O}_{2}{\;=\;2H}_{2}O$$

(6) $$H_{2}{+\;\mathrm{CO}+\;O}_{2}{\;=\;H}_{2}O+CO_{2}$$

(7) $${\mathrm{CH}}_{4}{+\;2O}_{2}{\;=\;\mathrm{CO}}_{2}{\;+\;2H}_{2}O$$

As SOFCs are not a heat engine, their efficiency is not limited by the Carnot cycle. However, such devices suffer from internal losses, especially at lower operating temperatures (T ≤ 800 °C). These losses are due to insufficient ionic conductivity and/or the slow kinetics of electrode processes. Therefore, traditional SOFCs containing YSZ as a supporting electrolyte and Ni-YSZ as an anode generally operate at temperatures around 900 °C, where the electrolyte’s electrical conductivity reaches a sufficient level (~0.1–0.2 S cm^−1^ [31,51]). On the other hand, lowering the temperature to 800 °C would simplify SOFC fabrication, reduce operating costs and extend their long-term lifespan. However, it is necessary to use other oxygen-conducting materials, since the ionic conductivity of YSZ strongly declines with cooling, reaching ~0.05 S cm^−1^ at 800 °C [16]. As electrolyte alternatives, compounds based on ceria (Ce_1-x_Gd_x_O_2-δ_, GDC, or Ce_1-x_Sm_x_O_2-δ_, SDC) and LSGM [38,39] can be used, since their conductivity at 800 °C is several times higher than that of YSZ [38]. From these compositions, it is possible to obtain sufficiently dense ceramics that are mechanically and thermally stable [31,32,33,57,58].

## 3. Anode Materials for SOFCs

Pure hydrogen, synthesis gas (Н_2_ + CО), simple hydrocarbons and ammonia can serve as fuel for SOFCs. The commercialization of SOFCs operating on hydrocarbon fuels has recently become promising due to the abundance of cheaper and easily stored natural gas. In this regard, the development of new anode materials is a key challenge for achieving efficient operations in the presence of methane, which can be used either directly (Reaction (4)) or after preliminary reforming to obtain syngas according to Equations (8)–(10):

(8) $${\mathrm{CH}}_{4}{\;+\;1/2O}_{2}{\;=\;\mathrm{CO}\;+\;2H}_{2}$$

(9) $${\mathrm{CH}}_{4}\;+\;H_{2}{O\;=\;\mathrm{CO}\;+\;3H}_{2}$$

(10) $${\mathrm{CH}}_{4}\;+\;CO_{2}{\;=\;2\mathrm{CO}\;+\;2H}_{2}$$

When using hydrocarbons, however, the problem of the carbonization of the anode material arises. During the direct supply of the methane-containing fuel (Reaction (4)), carbon deposition can occur due to cracking from a lack of oxygen ions (Equation (11)).

(11) $${\mathrm{CH}}_{4}\to C+2H_{2}$$

In a synthesis gas environment, the accumulation of carbon at the anode occurs due to the Boudouard reaction, which is a result of the high concentration of CO:

(12) $$2CO\to C+CO_{2}$$

and also due to the reduction of carbon monoxide with hydrogen:

(13) $$CO\;+\;H_{2}\to C+H_{2}O$$

Carbon can envelop electro-active anode particles and form carbon sediments both on the surface and in the bulk of the electrode material, causing its expansion and deactivation. To prevent the formation of a coke deposit, it is necessary to facilitate the electrochemical oxidation of CO on the surface of the anode (Equation (3)). Additionally, it is possible to supply some O_2_ to the feed gas, which will lead to CO oxidation or forced carbon burnout:

(14) $$2CO\;+O_{2}=2CO_{2}$$

(15) $$C+O_{2}=CO_{2}$$

Along with carbon accumulation, there is the problem of poisoning the anode materials with sulfur compounds, which are often present in small amounts in natural gas and can interact with electrode components. Moreover, sulfur prevents the reagents from accessing the electrode, blocking targeted electrochemical reactions.

Recognizing the prospects of using hydrocarbons in SOFCs, it is necessary to solve a number of problems in order to develop anode materials with a high level of stability and other important functional properties, including:

1. High catalytic activity in relation to fuel oxidation. In the case of hydrocarbon fuels, the catalytic properties of the material are preliminarily studied before using it as an anode. This is done to determine the mechanism and degree of fuel oxidation, synthesis gas yield and the possibility of carbon deposition after tests [36,41,42,45,47].
2. Sufficient electron conductivity. Electrons formed as a result of electrochemical reactions at anode/electrolyte interface must be transported to the external circuit not only through current collectors, but also through the supported anode with high conductivity to suppress any unwanted ohmic losses in the electrodes.
3. Thermal compatibility. The thermal expansion of the anode must be consistent with similar behavior in both the electrolyte and the current collector. This is required to prevent cracking between SOFC components during operation, heating, cooling or thermal cycling.
4. Chemical stability. The anode must be chemically stable at operating temperatures not only in oxidizing and reducing atmospheres, but also in relation to the electrolyte and the current collector. Otherwise, the resulting impurities can block the transfer of electrons or oxygen ions along the corresponding paths in the SOFCs. It should be noted that chemical stability must also be verified by sintering the SOFCs, where temperatures are higher compared to working temperatures.
5. Porosity. Since the fuel is a gas that must reach a triple-phase boundary (TPB) or the surface of a mixed-ionic conductor, the anode must exhibit a porous structure that retains its natural microstructural characteristics over a prolonged operating period.

Non-compliance to the listed requirements would kill the interest for application as anode materials.

The most common anode materials for SOFCs are porous Ni-YSZ cermets, in which nickel provides electrical conductivity and YSZ oxygen-ion conductivity. The main advantages of this composite include good chemical and thermal compatibility with YSZ electrolytes, high electrical conductivity and excellent electro-catalytic properties with respect to the oxidation of the hydrogen used as a fuel. However, there are some disadvantages: (i) instability of the microstructure during redox cycling, which causes the porous YSZ framework to lose mechanical strength due to the destruction of nickel–nickel contacts during reoxidation and reduction; (ii) the agglomeration of nickel particles using elevated temperatures and high current densities; such an agglomeration leads to the formation of isolated Ni-particles, with the subsequent loss of the electrical connection with the current collector [1]. In addition, the morphology and electrochemical activities of nickel-cermet composite electrodes strongly depend on the ratio and sizes of Ni and YSZ particles, as well as on the methods used to obtain the components [59,60].

Since the metallic nickel catalyzes a cracking reaction (Equation (11)), using Ni-YSZ ceramics in hydrocarbon fuels is difficult. This produces carbon fibers that can block pores and cause mechanical stress, with the subsequent destruction of the electrode and loss of SOFC performance [6,7,8]. In addition, nickel interacts with fuel impurities, especially sulfur. Even a very small amount of sulfur (as a part of H_2_S or S-containing organic compounds) poisons the catalyst, blocking electrochemically active zones and reducing SOFC performance. In this regard, many have sought either to improve cermets by introducing various additives (CaO, SrO [10]; Co, Fe, Cu [11]) and replacing YSZ with doped ceria [61] or to find new anode materials that can overcome the disadvantages of traditional cermets.

Perovskites with mixed ion-electronic conductivity (general formula ABO_3_) could be an alternative, since they can have an extended TPB compared to purely electronic or purely ionic conductors. In addition, oxide materials are good catalysts in hydrocarbon oxidation and can prevent carbon deposition due to the ability to exchange lattice oxygen with the gaseous phase [6,62].

To date, the existing data offer a wide range of perovskite anode materials with good performances in hydrocarbon fuels. The titanates (ATiO_3_) [63,64,65], chromites (ACrO_3_) [63], manganites (AMnO_3_) [63,66,67], ferrites (AFeO_3_) [63,68,69] and vanadates (AVO_3_) [70] of alkaline earth metals (A = Ca, Sr, Ba) are the best studied perovskites. Among double perovskites, strontium molybdates with general formula Sr_2_MMoO_6_ (M = Ni, Mg, Fe) have attracted particular interest over the last decade due to their ability to achieve the required combination of functional properties.

## 4. General Features of Sr_2_MMoO_6_ (M = Ni, Mg, Fe)

**Figure 2:**
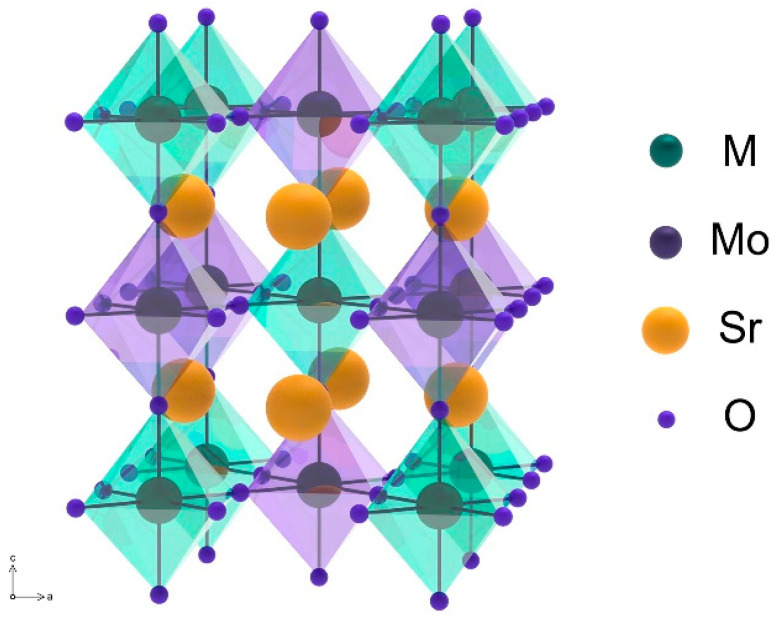
Cubic structure of double Sr_2_MMoO_6_ perovskites. Reproduced with permission [71]. Copyright 2006, Elsevier.

Double perovskites (Sr_2_MMoO_6_) have a modified perovskite structure (ABO_3_), where the BO_6_ and MoO_6_ octahedra are located in two alternating face-centered cubic sublattices described by the space group Fm3m (Figure 2). Factors involved in the MMo ordering are the differences in cation oxidation states and/or ionic radii. The octahedral voids are occupied by A-cations (Sr^2+^ in the present case).

**Figure 3:**
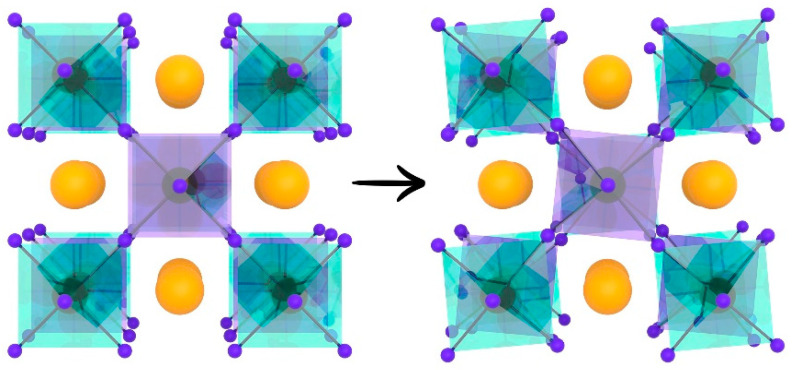
Structural phase transition of Sr_2_MMoO_6_ from cubic to tetragonal lattice associated with the rotation angle of the MO_6_ and MoO_6_ octahedra. Reproduced with permission [32]. Copyright 2003, Elsevier.

The cubic structure is usually distorted due to a mismatch between the sizes of Sr-, M- and Mo-cations, attaining the most energetically favorable form. When the BO_6_ and MoO_6_ octahedra rotate relative to each other by an angle, the value of which is determined by the displacement of oxygen atoms within the ab plane, the tetragonal lattice becomes more stable (Figure 3).

**Figure 4:**
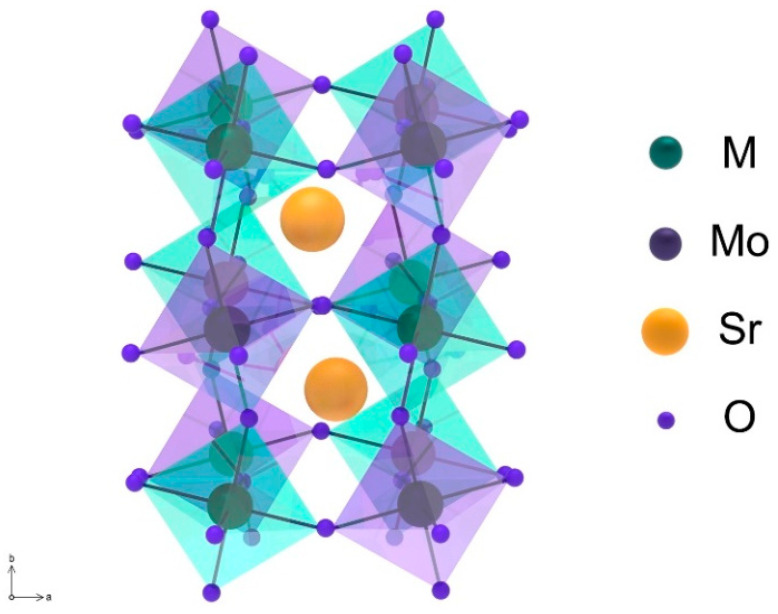
Monoclinic structure of Sr_2_MMoO_6_ formed during the octahedra tilting. Reproduced with permission [72]. Copyright 2013, AIP Publishing.

The double perovskite structure can also exhibit a lower degree of symmetry associated with octahedra tilting (Figure 4). In this case, monoclinic (a ≠ b ≠ c, α = β = 90°, γ ≠ 90°), triclinic (a ≠ b ≠ c, α ≠ β ≠ γ ≠ 90°) or orthorhombic (a ≠ b ≠ c, α = β = γ = 90°) polymorphs are formed.

The crystallographic structure of ABO_3_ (and A_2_BB’O_6_) perovskites can be predicted if we consider the discrepancy between the size of the A-site cations and the remaining space inside the oxygen octahedra. For this purpose, the value of the tolerance factor, t, is calculated, taking two A-O and B-O distances into account:

(16) $$t=\frac{r_{A}+r_{O}}{\sqrt{2}(r_{B}+r_{O})}$$

where r_A_, r_B_ and r_O_ are the effective ionic radii of A-, B- and O-ions, according to Shannon [73].

Excluding rare examples that arise due to the difficulty of determining variable oxidation states of the cations, the A_2_MMo’O_6_ compounds are found to be crystalized in various crustal structures [74]: hexagonal (space groups P6$\bar{3}$/mmc, P6$\bar{2}$c) at t > 1.05, cubic (Fm$\bar{3}$m) at 1.00 < t < 1.05, tetragonal at 0.97 < t < 1.00 and, finally, triclinic (P$\bar{1}$), monoclinic (P2_1_/n) or orthorhombic (Pmm2) at t < 0.97. For the double perovskites of Sr_2_NiMoO_6-δ_, Sr_2_MgMoO_6-δ_ and Sr_2_FeMoO_6-δ_, the theoretically calculated t values are 0.984, 0.977 and 0.963, respectively [20]. However, these often do not coincide with the experimentally calculated t values (Equation (17)). For example, in the following sections, it will be shown that the Sr_2_MgMoO_6-δ_ and Sr_2_FeMoO_6-δ_ compounds can be also formed in triclinic and tetragonal systems.

(17) $$t=\frac{d_{A-O}}{\sqrt{2}d_{B-O}}$$

where d_A-O_ and d_B-O_ are the average distances between corresponding atoms.

From the viewpoint of defect chemistry, the Sr_2_MMoO_6-δ_ perovskites exhibit commonalities: the presence of a Mo^6+^/Mo^5+^ redox pair and a certain amount of oxygen vacancies formed according to Equation (18). In addition, the MO_6_ octahedra in the Sr_2_MMoO_6-δ_ oxides determine their electronic properties, as, for instance, the ordering between Mg^2+^ and Mo^6+^ cations is hindering the charge delocalization on the M-O-Mo network, whereas charge carrier exchange between Fe^2+^/Fe^3+^ and Mo^6+^/Mo^5+^ is a charge carrier delocalization promoter. Therefore, replacing the B-position elements also allows for the modification of their structural, thermal, electric transport, catalytic and other functional properties.

(18) $$2Mo_{Mo}^{x}+O_{O}^{x}⇄2M{o^{\prime }}_{Mo}+V_{O}^{••}+1/2O_{2},$$

where $V_{O}^{••}$ is the oxygen vacancy, $Mo_{Mo}^{x}$ is the Mo^6+^-ion (oxidized state) and $M{o^{\prime }}_{Mo}$ is the Mo^5+^-ion (reduced state).

For non-composite (i.e., single-phase) anode materials, oxygen vacancies are critical for realizing oxygen-ion transport. The Mo^+6^/Mo^+5^ redox pairs also provide for electron transport in case of the absence of other transition elements. The ability of these materials to prevent carbon deposition is due to carbon interaction with the lattice oxygen [6,62].

Analyzing the data from the literature, it can be noted that the most promising and studied molybdates are strontium molybdates, where the M-position is occupied by nickel, magnesium and iron. The SOFCs based on these molybdates and LSGM electrolytes yield very high power densities (Table 1).

In Table 1 the power density values for the traditional Ni-YSZ cermet anodes are also presented. From this comparison one can see that molybdates Sr_2_MMoO_6-δ_ (M = Ni, Mg, Fe) are not inferior to the traditional anode in terms of power densities. Moreover, in case of the Ni-cermet anodes, the carbon particles formed during hydrocarbon fuel pyrolysis are deposited on the electrode surface that leads to the cell degradation [82]. The problem of coke formation may be solved by incorporating a catalyst (Au, Pd, Ru) into the Ni-based cermet anodes to avoid the conditions of coke formation. Obviously, it will increase the cost of the Ni-cermet anodes, which does not seem to be economically viable. The investigations of carbon deposition behaviors of the Ni-YSZ-based anodes after treatment in biogas [83] and the Sr_2_MnMoO_6-δ_/NiO-Ce_0.8_Sm_0.2_O_1.9_ composite in methane [28] illustrate the undoubted advantages of the molybdate-based anodes.

The electrochemical characteristics of anode materials largely depend on their functional properties: stability in a fuel gas environment, electrical conductivity and chemical and thermal compatibility with electrolyte materials. Therefore, in the following sections, the main emphasis will be devoted to summarizing the results achieved for Sr_2_NiMoO_6-δ_, Sr_2_MgMoO_6-δ_, Sr_2_FeMoO_6-δ_ and Sr_2_Fe_1.5_Mo_0.5_O_6-δ_.

## 5. Functional Properties of Sr_2_MMoO_6_ (M = Ni, Mg, Fe)

The present section, aiming at properties of the Sr_2_MMoO_6-δ_ phases, has the following common structure of description: preparation, crystal features, thermodynamic stability, thermal and electrical properties, and chemical compatibility with the state-of-the-art electrolyte materials.

### 5.1. Sr_2_NiMoO_6-δ_

The double perovskite Sr_2_NiMoO_6-δ_ has been considered as the basis for promising anode materials, since the corresponding SOFCs show quite high levels of performance (Table 1). This compound can be easily obtained in a single-phase form in air using various techniques, including the easy and simple method of solid state synthesis [71,84,85,86,87]. The starting materials of SrCO_3_, NiO, and MoO_3_ are mixed and ground together over a long period with subsequent annealing. The final annealing temperatures are rather high in the case of solid state synthesis: 1300 °C for 6 h [84], 1250–1350 °C for 12 h [86] or 48 h [71,87].

The most widely used method for synthesizing Sr_2_NiMoO_6-δ_ is sol-gel technology [24,34,39,75,88,89]. A crystalline hydrate of ammonium heptamolybdate (NH_4_)_6_Mo_7_O_24_·4H_2_O is used as an Mo-containing component; together with Sr(NO_3_)_2_ and Ni(NO_3_)_2_∙6H_2_O, this is dissolved in water. In some works, strontium carbonate (SrCO_3_) and nickel oxide (NiO) are used instead of nitrates for the dissolution of which nitric acid is required [88]. Ethylenediaminetetraacetic acid (EDTA) is added to the prepared solution as a chelate agent and then a pH (~7) is tailored with an aqueous ammonia solution. The resulting mixture is converted into a sol during the heat treatment, and then into a gel, which is subsequently dried and calcined. The calcination is usually carried out with two steps: first at 400 °C to remove organic residues, and then at higher temperatures, which can vary greatly: at 1250 °C for 24 h [24], at 800°C for 10 h [34], at 1300 °C for 24 h [39] or at 1000 °C for 12 h [88] in air.

The citrate-nitrate method was used in [90] to synthesize the complex oxide Sr_2_NiMoO_6-δ_; this method consists of the thermolysis of a mixture of nitrates and citric acid, which acts as both a chelating agent and fuel. Thermal treatment was conducted in the same way as in EDTA. The resulting powder was calcined at 850 °C for 12 h. After the synthesis, the sample contained a small amount of a SrMoO_4_ impurity phase (which remained even after sintering in air at 1350 °C for 12 h).

In work [91], the authors used the lyophilization of an aqueous solution of cations to obtain Sr_2_NiMoO_6-δ_. Sr(NO_3_)_2_ and Ni(NO_3_)_2_∙6H_2_O were dissolved in water, while MoO_3_ was dissolved in dilute nitric acid, with EDTA and ammonia added subsequently. The resulting solution was frozen dropwise to liquid nitrogen. The frozen drops were dehydrated by vacuum sublimation in a freeze dryer for 2 days until an amorphous state was formed. It was then heated three times: at 300 °C to burn organic residue, at 800 °C to remove carbon-containing particles and at 1200 °C for 1 h until crystallization was achieved.

The crystal lattice of Sr_2_NiMoO_6-δ_ is described within the framework of a tetragonal system with the space group of I4/m [71,87,88,90,91,92] and lattice parameters of *a* = 5.540 Å, *c* = 7.890 Å [71,87,88]. This compound is characterized by the presence of a second-order transition from the tetragonal I4/m to the cubic Fm3m structure when temperature increases. This transition is associated with the rotation of the NiO_6_ and MoO_6_ octahedra (Figure 3) and takes place at a temperature of 235 °C [92], 250 °C [91] or 277 °C [87].

**Figure 5:**
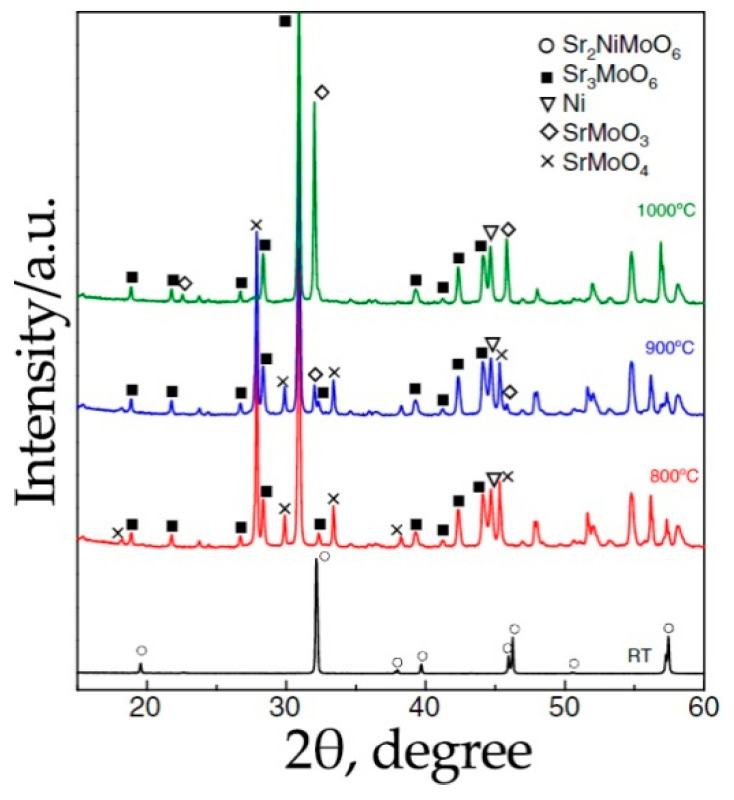
XRD patterns recorded for Sr_2_NiMoO_6-δ_ calcined in 5%H_2_/Ar at different temperatures for 24 h. Reproduced with permission [91]. Copyright 2013, Elsevier.

The stability of Sr_2_NiMoO_6-δ_ under reducing conditions was studied in [24,34,91]. According to X-ray diffraction (XRD) data [34], this material is single phase after sintering at 1200 °C in a forming 5%H_2_/N_2_ gas (here and below, the volume units are used). However, energy dispersive X-ray spectroscopy analysis showed the presence of metallic nickel nanoparticles. The authors of [24] have shown that Sr_2_NiMoO_6-δ_ decomposes in an atmosphere of 5%H_2_/Ar above 800 °C. This is in agreement with other data [91], which show the complete decomposition of Sr_2_NiMoO_6-δ_ into Sr_3_MoO_6_, SrMoO_3_, SrMoO_4_ and Ni after prolonged treatment of the double perovskite in an atmosphere of 5%H_2_/Ar (Figure 5). In pure CO_2_, double perovskite is destroyed at 600 °C; in this case, the formation of SrCO_3_ and SrMoO_4_ impurity phases occurs [91].

The mentioned phase relation for Sr_2_NiMoO_6-δ_ in reducing atmospheres might be explained by a low redox ability of Ni-ions. The latter are exsolved from the perovskite structure until the formation of metallic Ni-particles and unstable “Sr_2_MoO_6_” residue that decomposes into a number of more simple molybdates.

The thermal expansion coefficients of Sr_2_NiMoO_6-δ_ in air (Table 2) are in agreement with similar parameters for LSGM materials (11.4 × 10^−6^ K^−1^ for LSGM [93,94,95]).

The values of the electrical conductivity of Sr_2_NiMoO_6-δ_ (Table 3) are low enough for practical use in SOFCs. The high conductivity achieved in [75] is probably associated with the formation of highly conductive impurity phases (Ni and SrMoO_3_ in pure hydrogen at 850 °C).

The revealed varieties in structure, stability and functional properties of the same material (Sr_2_NiMoO_6-δ_) can be explained by its pre-history. As shown in works [25,84], the synthesis methods of the Sr_2_MMoO_6-δ_ compounds determine their phase compositions, crystal structures, microstructural morphologies, and physico-chemical properties; in particular, electrical transport properties and thermodynamic stability can be considerably varied. This comes from the fact that the preparation pathways and the synthesis/sintering conditions of Sr_2_MMoO_6-δ_ determine the content of Mo^+5^ ions (Equation (18)) in the obtained phases [75]. As a result, a high content of Mo^+5^ ions in Sr_2_MMoO_6-δ_ governs stability of the final oxides in reducing atmospheres [25] and high values of electrical conductivity [75]. Therefore, when characterizing the structure and properties of Sr_2_MMoO_6-δ_, their preparation details should be thoroughly analysed.

**Figure 6:**
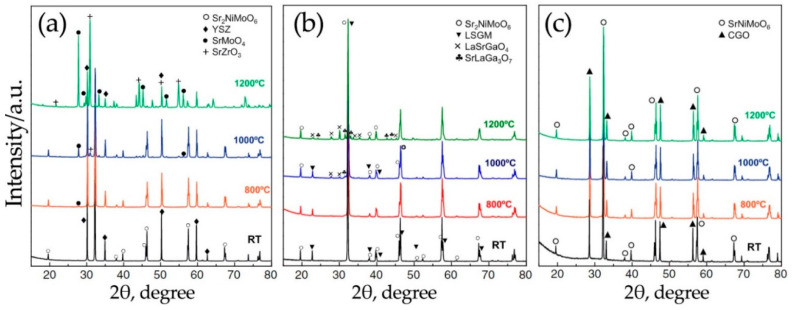
XRD patterns recorded for the as-prepared and calcined mixtures composed of: Sr_2_NiMoO_6-δ_ and yttria-stabilized zirconia (YSZ) (**a**), Sr_2_NiMoO_6-δ_ and LSGM (**b**) and Sr_2_NiMoO_6-δ_ and Ce_1-x_Gd_x_O_2–δ_ (GDC) (**c**). Reproduced with permission [91]. Copyright 2013, Elsevier.

Proposing Sr_2_NiMoO_6-δ_ as an alternative anode material, most authors recommend using it in combination with LSGM [24,75], CGO [34] or CSO [39] electrolytes. However, the temperature of sintering the anode suspension to the LSGM electrolyte should not exceed 1000 °C. On the contrary, they interact chemically with each other [91], leading to the formation of poorly conducting impurity phases, LaSrGaO_4_ and SrLaGa_3_O_7_ (Figure 6). Sr_2_NiMoO_6-δ_ does not react with oxide-based materials (CGO and CSO) even at 1200 °C; therefore, CGO and CSO can also be used as protective layers between the LSGM electrolyte and the anode, formed at temperatures above 1000 °C. According to [82,91,96], the double perovskite molybdates interact with YSZ electrolytes with the formation of SrMoO_4_ (at temperatures above 800 °C) and SrZrO_3_ (at temperatures above 1000 °C) phases. For this reason, Sr_2_MMoO_6_ cannot be considered as anode material for high-temperature SOFCs.

**Figure 7:**
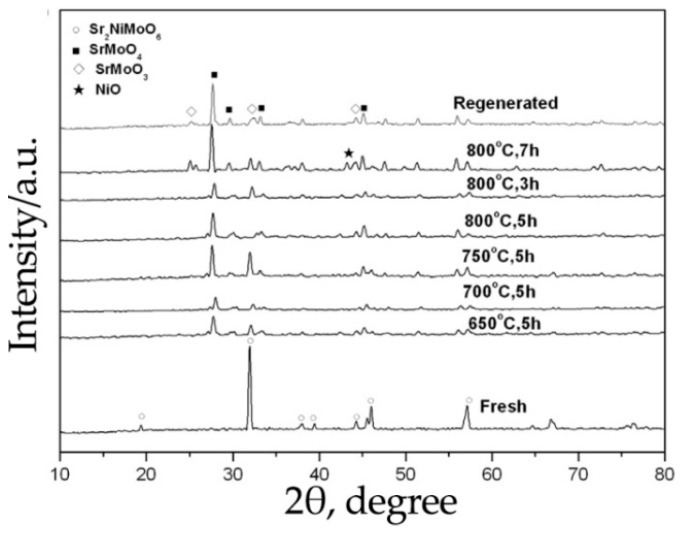
XRD patterns recorded for Sr_2_NiMoO_6-δ_ after its calcination in 0.1%H_2_S/Ar at different temperatures. Reproduced with permission [39]. Copyright 2015, Elsevier.

The chemical stability of Sr_2_NiMoO_6-δ_ in hydrocarbon fuels has been insufficiently discussed in the literature. Some works indicate that this material is unstable in a methane atmosphere and in conditions containing sulfur. In [39], it was reported that this perovskite is unstable in a 0.1%H_2_S/H_2_ environment, even at 650 °C (Figure 7). In addition, with an increase in temperature in this medium, the grain boundaries of the sintered Sr_2_NiMoO_6-δ_ ceramic were covered with needles and filamentous inclusions, which may be related to metal sulfide(s). Upon testing the SOFC, the Sr_2_NiMoO_6-δ_ anode was identified to be chemically unstable when methane CH_4_ was supplied as a fuel [34]. In addition, an undesirable carbonization reaction (Equation (11)) was also detected.

### 5.2. Sr_2_MgMoO_6-δ_

There is not a single comprehensive study on the properties of the Sr_2_MgMoO_6-δ_ oxide. Separate works have been devoted to either electric transport or thermal properties or the design and testing of a fuel cell. The results obtained are quite distinct, which can be explained by the conditions of synthesis and the subsequent sintering of the material, which affects the phase composition, density, microstructure, and consequently other properties.

**Figure 8:**
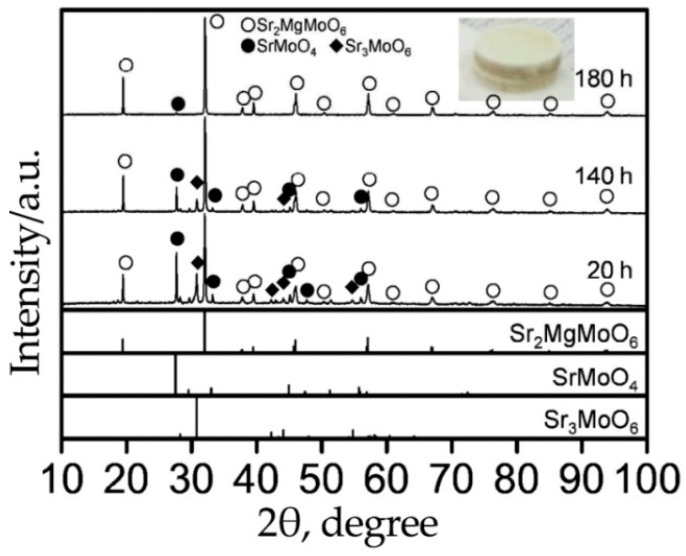
XRD patterns recorded for Sr_2_MgMoO_6-δ_ calcined in air at different exposure times. Reproduced with permission [98]. Copyright 2017, John Wiley & Sons.

As Sr_2_NiMoO_6-δ_, Sr_2_MgMoO_6-δ_ can be obtained through different synthesis methods: solid state synthesis [19,97,98,99,100,101,102,103], solution methods (including sol-gel technology using citric acid [24,37,45,72,104,105,106,107] and EDTA [20,76,77,108,109]), the combustion method using glycine as a fuel and complexing agent [110,111] and the freeze-drying method [88,108]. The main disadvantage of this compound is its non-single phase after synthesis in air (Figure 8). Therefore, in almost all works, Sr_2_MgMoO_6-δ_ was additionally treated in 5%H_2_/inert gas (Ar or N_2_) at elevated temperatures. The temperature and exposure time varied from 1000 °C to 1300 °C and from 10 to 40 h, respectively. Sr_2_MgMoO_6-δ_ was obtained in a single-phase form in air only in [104] after annealing at 1450 °C for 10 h and in [111] at a certain fuel and oxidizer ratio with final annealing at 1000 °C for 6 h.

It should also be noted that Sr_2_MgMoO_6-δ_ does not decompose (in contrast to Sr_2_NiMoO_6-δ_) in H_2_-containing atmospheres at high temperatures. However, it is also unstable in a CO_2_ environment at 600 °C [91].

Discussing the crystal structure of Sr_2_MgMoO_6-δ_, the compound can exhibit cubic (Fm3m [89]), tetragonal (I4/m [19,72,97,98,106,112,113]), monoclinic (P2 [37], P21/n [77,109]) or triclinic I-1 [101,103,104,108,111] crystal structures, depending on the synthesis methods. The triclinic structure was proved by neutron diffraction analysis [101]. According to [108], the cell parameters for the triclinic Sr_2_MgMoO_6-δ_ at room temperature were: *a* = 5.5702 Å, *b* = 5.5709 Å, *c* = 7.9228 Å, α = 89.96°, β = 90.01°, γ = 90.00°. The phase transitions of Sr_2_MgMoO_6-δ_ were studied in [72,108]. It was reported that this material undergoes a structural phase transition from the tetragonal (I4/m) to the cubic (Fm3m) structure at 300 °C [72], or from the triclinic (I-1) to the cubic (Fm3m) structure with cubic cell parameter *a* = 7.9308 Å at 250 °C [108].

Table 4 and Table 5 list electrical and thermomechanical properties of Sr_2_MgMoO_6-δ_. The functional properties of this compound are closely dependent on the preparation method and the reduction degree. An acceptable value of electrical conductivity was obtained in [100] when synthesizing the sample via solid state synthesis, followed by firing in 5%H_2_/N_2_ in two stages, both at 1300 °C for 4 h. The lower conductivity values obtained in [77,97,104,113] might be associated with the fact that Sr_2_MgMoO_6-δ_ was subject to reduction at temperatures from 800 °C to 1200 °C. These conditions probably lead to the incomplete reduction of the molybdate; as a consequence, an insignificant concentration of Mo^+5^-ions was achieved (Equation (18)).

The thermal behavior for Sr_2_MgMoO_6-δ_ was studied only in air, despite the fact that this material was synthesized in a hydrogen atmosphere and that it becomes non-single phase under oxidizing conditions. More precisely, the impurity phases of SrMoO_4_ and Sr_3_MoO_6_ are formed along with the target Sr_2_MgMoO_6-δ_ compound after its synthesis in an air atmosphere [98]. Although Mg-ions exhibit very high chemical stability due to the constant oxidation state (+2), they cannot compensate for an excess of the lattice oxygen upon the oxidation of Sr_2_MgMoO_6-δ_, leading to phase decomposition.

**Figure 9:**
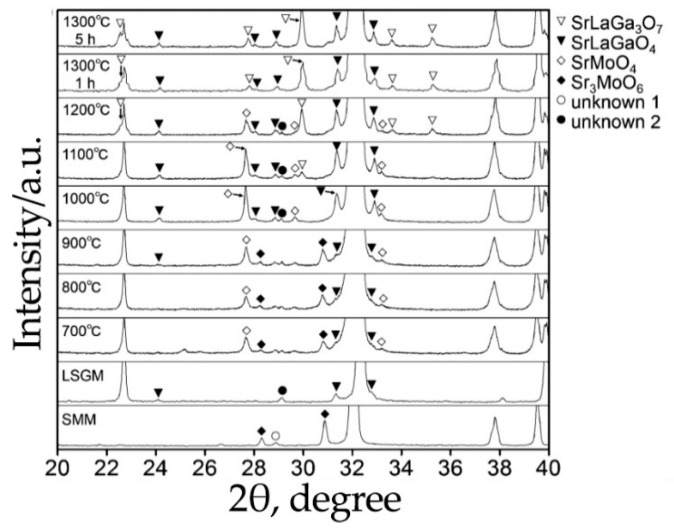
XRD patterns recorded for mixtures of Sr_2_MgMoO_6-δ_ and LSGM calcined in air at different temperatures. Reproduced with permission [99]. Copyright 2018, The Ceramic Society of Japan.

The thermal compatibility of Sr_2_MgMoO_6-δ_ with an LSGM electrolyte was studied in [99]. It was found that an insignificant interaction between these components begins after annealing at 700 °C (Figure 9). In this regard, a protective layer of doped ceria must be applied between the anode and the electrolyte when designing SOFCs. However, in most of the works devoted to the testing of SOFCs, no protective layers were used [76,77].

**Figure 10:**
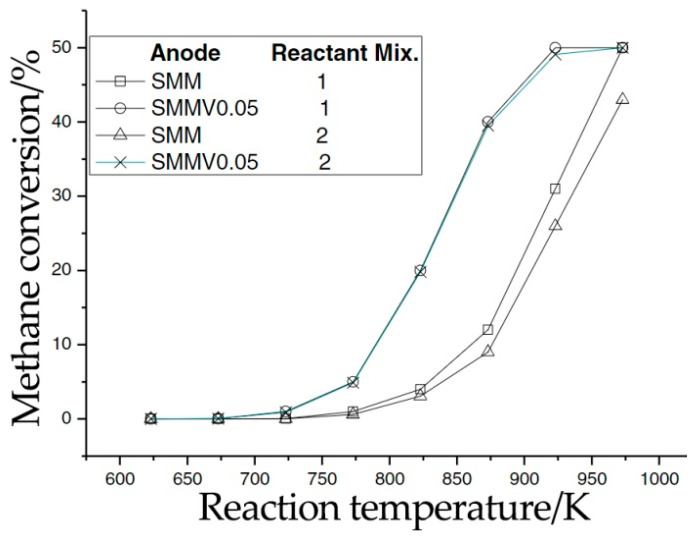
Temperature dependence of the conversion degree of biogas in the case of using Sr_2_MgMoO_6-δ_ (SMM) or Sr_2_MgMo_0.95_V_0.05_O_6-δ_ (SMMV0.05): 1–6% CH_4_, 6% O_2_, 4% CO_2_, 84 % N_2_; 2–6% CH_4_, 6% O_2_, 4% CO_2_, 83 % N_2_, 1% H_2_S. Reproduced with permission [45]. Copyright 2015, Elsevier.

Particular attention has been paid to Sr_2_MgMoO_6-δ_ due to its good catalytic properties in the oxidation of hydrocarbons and acceptable tolerance with regards to carbonization and sulfur poisoning. For example, Sr_2_MgMo_1-x_V_x_O_6−__δ_ (x = 0–0.2) materials were verified in catalytic tests in biogas [45]. Two types of mixtures were fed into a reactor at temperatures of 300–600 °C: 6% CH_4_, 6% O_2_, 4% CO_2_, balanced with N_2_ and 6% CH_4_, 6% O_2_, 4% CO_2_, 1% H_2_S balanced with N_2_. After isothermal holding, CO_2_, H_2_O and a small amount of SO_2_ were detected as reaction products for the reaction mixture with 1% H_2_S; the methane conversion rate reached 50% (Figure 10). According to the calculation of the sulfur balance, apart from sulfur oxide SO_2_ and hydrogen sulfide H_2_S, no compounds were formed, which proves the stability of Sr_2_MgMoO_6-δ_ with regards to sulfur poisoning. The phase stability of Sr_2_MgMoO_6-δ_ in a 10%CН_4_/Ar medium was proved using the XRD analysis [109]: no traces of carbon were found during testing this sample in comparison to an Ni-GDC ceramic taken as a blank sample.

The effect of sulfur poisoning on the SOFC performance of a fuel cell with a Sr_2_MgMoO_6-δ_ anode was studied in [37]. After feeding a mixture containing 100 ppm H_2_S in H_2_ at 800 °C for 90 h, the anode material remained in its single phase, but the performance of the SOFC decreased, which was attributed to the accumulation of sulfur on the buffer layer. The high sulfur tolerance of Sr_2_MgMoO_6-δ_ was also reported in other works [77,113,114].

### 5.3. Sr_2_FeMoO_6-δ_

**Figure 11:**
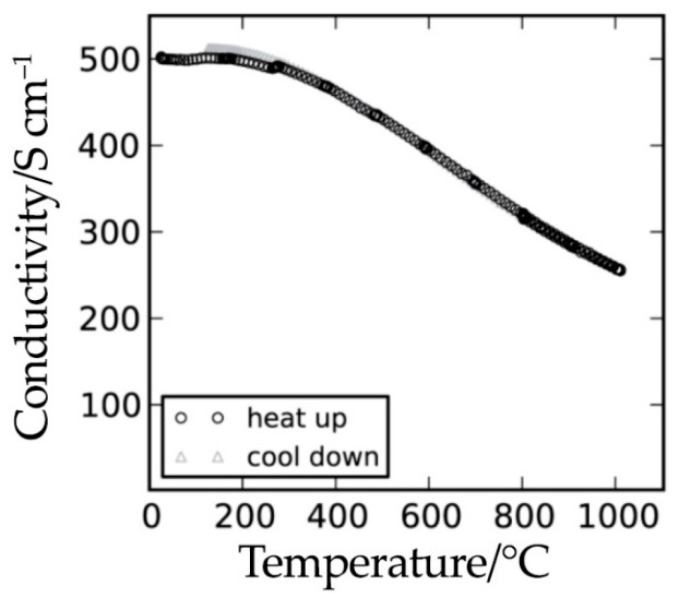
Conductivity of Sr_2_FeMoO_6-δ_ in 9%H_2_/Ar. Reproduced with permission [18]. Copyright 2010, The Electrochemical Society.

Iron-containing molybdates have attracted increased attention due to the presence of Fe-Mo pairs, in which a mixed valence is characteristic of both the molybdenum (Mo^5+^/Mo^6+^) and iron (Fe^2+^/Fe^3+^) ions. This peculiarity causes high electron transport. For example, the complex Sr_2_FeMoO_6-δ_ oxide, having a metallic type of conductivity (Figure 11), is very different from the other double perovskites. The conductivity values for a given compound in a reducing atmosphere vary in a wide range, from 100 to 300 S cm^−1^ at 800 °C [18,115].

The Sr_2_FeMoO_6-δ_ complex oxide can be prepared in the same manner as the previous Ni- and Mg-containing systems. Sr_2_FeMoO_6-δ_ was synthesized by solid state synthesis in [18,19,78]. A mixture of the starting reagents (SrCO_3_, Fe_2_O_3_, and MoO_3_) was first calcined in air and then in a 5%H_2_/Ar gas at 1100 °C for 10 h [78], 30 h [18] or at 1250 °C for 12 h [18]. Solution methods were used in [40,47,114,115,116,117]. (NH_4_)_6_Mo_7_O_24_·4H_2_O, Sr(NO_3_)_2_ and Fe(NO_3_)_3_·9H_2_O were used as starting salts, which were dissolved in water. EDTA, citric acid and ammonia were added in sequence. After the evaporation of the resulting solution and spontaneous combustion, the obtained powder was annealed first at low temperatures (300–400 °C) to remove organic components and then at higher temperatures. The final heat treatment was carried out in 5%H_2_/Ar at 1100 °C for 20 h [40], 24 h [116] or 2 h [47]. In [117] the finishing powder was heat-treated in air and atmosphere of 5%H_2_/N_2_ for 3 h at 800 °C. Similar to the double perovskite Sr_2_MgMoO_6-δ_, the complex Sr_2_FeMoO_6-δ_ oxide is highly stable in a hydrogen atmosphere, while in air it tends to decompose. According to [116,118] the Sr_2_FeMoO_6-δ_ oxide remains a single phase in the pO_2_ range 10^−12^–10^−14^ atm. Chemical stability of Sr_2_FeMoO_6-δ_ as a representative of a Sr_2_Fe_1-x_Mo_x_O_6-δ_ family [116,118,119] is caused by the existence of two redox active elements showing a variety in their oxidation states (+2, +3 and +4 for iron and +5 and +6 for molybdenum). In reducing atmospheres, these cations exist in reduced states (Fe^+2^, Fe^+3^, Mo^+5^, Mo^+6^), adjusting the oxygen content below 6.0 (i.e., δ > 0). In atmospheres with high oxygen partial pressures, the content of oxidized cations (Fe^3+^, Fe^4+^, Mo^6+^) increase, leading to unstable over-stoichiometry products decomposed until the formation of a SrMoO_4_ impurity.

The crystal structure of Sr_2_FeMoO_6-δ_ is described by tetragonal symmetry with the space group I4/m [19,78,113], Р4/mmm [116], or I4/mmm [47]. Manasa et al. [115] reported that this material has a cubic unit cell (sp. gr. Fm3m) after reduction and a tetragonal unit cell (sp. gr. I4/m) after annealing in air. The parameters for the tetragonal structure were refined in [116]: *a* = *b* = 5.575 Å, *c* = 7.907 Å; and in [47]: *a* = *b* = 5.564 Å, *c* = 7.888 Å. Possessing high electrical conductivity (nearly 100 S cm^−1^ in the temperature range 25–827 °C), the double Sr_2_FeMoO_6-δ_ perovskite also demonstrates quite acceptable TEC values (13.7(4)·10^−6^ K^−1^) in N_2_ and 5%H_2_/Ar atmospheres [40,78,116]. However, its thermal behavior in air remains unstudied.

**Figure 12:**
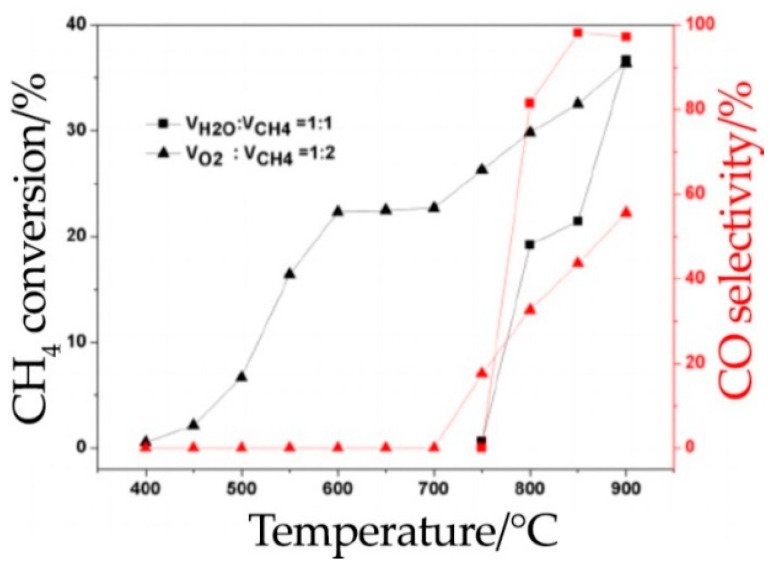
Catalytic activity of Sr_2_FeMoO_6-δ_. Reproduced with permission [47]. Copyright 2018, John Wiley & Sons.

Research on the stability of the Sr_2_FeMoO_6-δ_ anode material in hydrocarbon fuel was carried out in [40,78,114]. It was shown that an SOFC consisting of Sr_2_FeMoO_6-δ_|La_0.8_Sr_0.2_Ga_0.83_Mg_0.17_O_3-δ_|Ba_0.5_Sr_0.5_Co_0.8_Fe_0.2_O_3-δ_ operated stably when methane was supplied at 850 °C, and the maximal specific power densities decreased only by 5% after the 20th cycle [40]. The catalytic activity of Sr_2_FeMoO_6-δ_ during methane oxidation with oxygen was investigated in [35,47]. It was found that methane is completely oxidized according to Reaction (7) at a high conversion rate, reaching 50% at 750 °C [35]. In cases of partial oxidation (reaction when a CН_4_/О_2_ mixture is fed in a 1:1 ratio), the methane is completely oxidized according to Equation (10), while the conversion rate reaches 50% at 750 °C [35]. In cases of partial oxidation (Equation (8)) when using a mixture of CН_4_/О_2_ at a ratio of 2:1, a maximal conversion degree of 36.6% was achieved at 900 °C with a corresponding CO selectivity of 97.2% (Figure 12) [47].

### 5.4. Sr_2_Fe_1.5_Mo_0.5_O_6-δ_

The most studied iron-containing molybdate is Sr_2_Fe_1.5_Mo_0.5_O_6-δ_ with an oxygen nonstoichiometry level (δ) of 0.10 [116]. Oxygen vacancies in this material are mainly transported through the Fe-O-Fe bonds instead of the Mo-O-Fe and Mo-O-Mo bonds; at the same time, the Fe-O bonds are relatively weak, which contributes to a high level of oxygen conductivity. This material has attracted significant attention due to its stability in both oxidizing and reducing atmospheres, as well as the possibility of using it not only as an anode, but also as a cathode [120,121,122].

In most works, Sr_2_Fe_1.5_Mo_0.5_O_6-δ_ was synthesized by dissolving the initial salts ((NH_4_)_6_Mo_7_O_24_·4H_2_O, Sr(NO_3_)_2_ and Fe(NO_3_)_3_·9H_2_O) in water, adding glycine and citric acid and annealing the precursors obtained after spontaneous combustion at 950–1100 °C in air [21,80,122,123,124,125,126,127,128,129,130,131,132]. This material was also obtained via solid state synthesis in [102,133,134].

The double perovskite Sr_2_Fe_1.5_Mo_0.5_O_6-δ_ has a cubic face-centered (Fm3m) [80,127,133,135,136] or primitive (Pm3m) [21,91,122] crystal structure after sintering in air. The lattice parameters (*a* = *b* = *c*) calculated within the Fm3m space group are 7.852 Å [127], 7.860 Å [80], 7.845 Å [135], 7.349 Å [136], 7.843 Å [133]; for the Pm3m space group, they are equal to 3.928 Å [21]. A tetragonal structure with an I4/mcm space group was proved by neutron diffraction analysis in [137]; when Sr_2_Fe_1.5_Mo_0.5_O_6-δ_ was heated in 5%H_2_/Ar, it underwent phase transformations into a cubic system (sp. gr. Pm3m). However, it reverted to a tetragonal system after cooling. It is interesting that in the structure of the Sr_2_Fe_1.5_Mo_0.5_O_6-δ_ perovskite, the iron and molybdenum atoms in a ratio of 3:1 are distributed randomly: iron-molybdenum ordering stops. Perhaps this is the reason why Sr_2_Fe_1.5_Mo_0.5_O_6-δ_ differs strongly from other double perovskites in terms of its properties, as it is no longer a double perovskite but a simple one and should be rather formulated SrFe_0.75_Mo_0.25_O_3-δ/2_.

**Figure 13:**
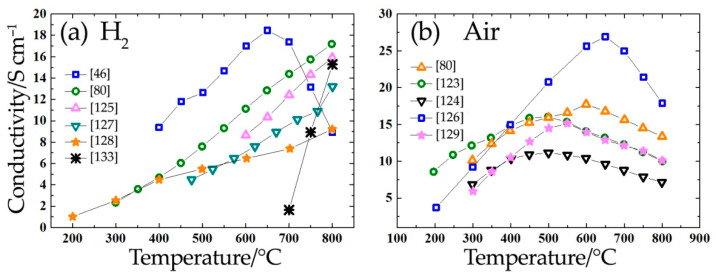
Conductivity values of the Sr_2_Fe_1.5_Mo_0.5_O_6-δ_ ceramic in reducing (**a**) and oxidizing (**b**) atmospheres.

When analyzing the electrical transport properties of Sr_2_Fe_1.5_Mo_0.5_O_6-δ_, different authors have produced quite distinct results. The electrical conductivity values vary from 9 to 310 S cm^−1^ in hydrogen and from 10 to 550 S cm^−1^ in air (Figure 13, Table 6). One of the requirements for anode materials [50] is their high electronic conductivity for effective electrical connectivity with interconnectors. From Table 3 and Table 5 and Figure 13a, it can be concluded that the electrical conductivity of the Sr_2_MMoO_6_ (M = Ni, Mg, Fe) molybdates are below the required values (10 S cm^−1^) in reducing atmospheres at low temperatures; therefore, they can be used for intermediate-temperature SOFCs.

A similar disagreement also occurs when considering the thermal properties (Table 7): some authors gave TEC values calculated over the entire temperature range, while others identified several sections in the dilatometric curve with different slopes. It should be noted that the TEC values are rather high compared to electrolyte materials, which could be a significant drawback from application viewpoints.

**Figure 14:**
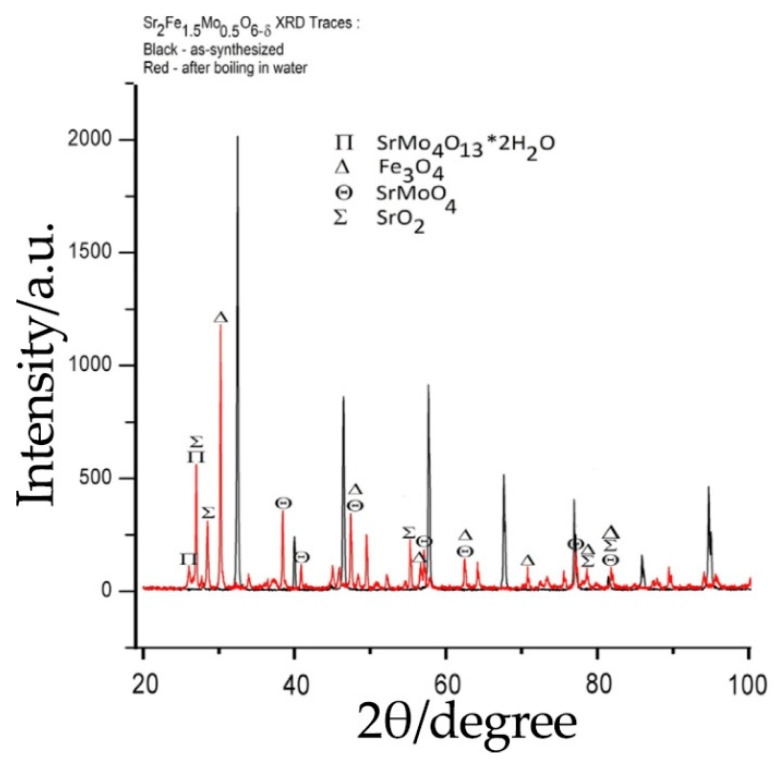
XRD patterns of Sr_2_Fe_1.5_Mo_0.5_O_6-δ_ before and after its treatment in a boiling water. Reproduced with permission [128]. Copyright 2013, Elsevier.

The chemical stability of Sr_2_Fe_1.5_Mo_0.5_O_6-δ_ in various atmospheres has been widely studied. According to the data from the literature, this is stable in a hydrogen atmosphere even after annealing at 1000 °C for 24 h [91,135] and in pure CO_2_ at 800 °C [91]. However, there is a significant drawback: the sample is unstable in humid atmospheres at low temperatures. The authors of [128] noted that this double perovskite easily reacts with water to form strontium hydroxide Sr(OH)_2_, decomposing into Fe_3_O_4_, SrMoO_4_ and SrO_2_ (Figure 14). It was noted that upon repeated heating to 800 °C, the formed impurities could again react with each other, forming Sr_2_Fe_1.5_Mo_0.5_O_6-δ_. This behavior is extremely undesirable for the real application of this molybdate in SOFCs, since the corresponding anode can decompose in a humid environment when heated or cooled, causing possible mechanical stress between the anode and electrolyte.

**Figure 15:**
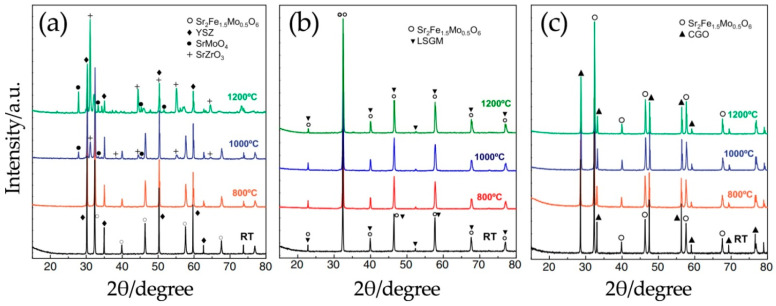
XRD patterns of different mixtures at room temperature and after high-temperature treatment: Sr_2_Fe_1.5_Mo_0.5_O_6-δ_ and YSZ (**a**), Sr_2_Fe_1.5_Mo_0.5_O_6-δ_ and LSGM (**b**) Sr_2_Fe_1.5_Mo_0.5_O_6-δ_ and GDC (**c**). Reproduced with permission [91]. Copyright 2013, Elsevier.

The chemical compatibility of Sr_2_Fe_1.5_Mo_0.5_O_6-δ_ with state-of-the-art electrolyte materials was studied in detail in [91]. It was found that Sr_2_Fe_1.5_Mo_0.5_O_6-δ_ does not react with Ce_0.8_Gd_0.2_O_2–δ_ even at 1200 °C; interaction with the YSZ electrolyte begins at 1000 °C. The study of chemical compatibility with LSGM is difficult because the diffraction peaks of the two phases overlap. However, a noticeable shift in their position was not observed at different sintering temperatures, and no impurity phases were found (Figure 15).

**Figure 16:**
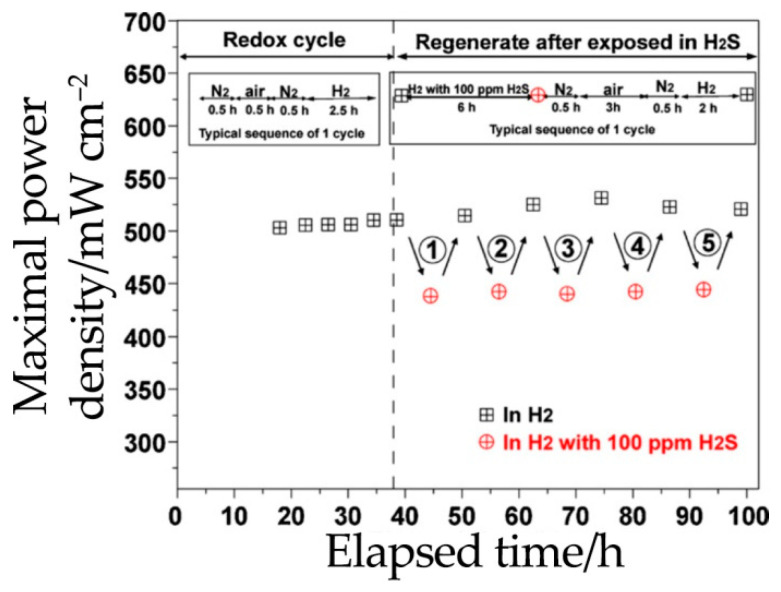
Short-term stability of the Sr_2_Fe_1.5_Mo_0.5_O_6-δ_|LSGM|Sr_2_Fe_1.5_Mo_0.5_O_6-δ_ (SFM|LSGM|SFM) cell at 800 °C during a number of cycles: the first five cycles are redox changes, while other five correspond to a gradual replacement of H_2_ with 100 ppm H_2_S/H_2_. Reproduced with permission [21]. Copyright 2011, Elsevier.

The tolerance of the Sr_2_Fe_1.5_Mo_0.5_O_6-δ_ phase to a fuel containing sulfur was investigated in [21,46,137,138]. In work [46], during testing of an SOFC based on the Sr_2_Fe_1.5_Mo_0.5_O_6-δ_-Ce_0.9_Gd_0.1_O_2–δ_ composite anode (Sr_2_Fe_1.5_Mo_0.5_O_6-δ_-Ce_0.9_Gd_0.1_O_2-δ_|Ce_0.9_Gd_0.1_O_2-δ_|La_0.6_Sr_0.4_Co_0.2_Fe_0.8_O_3-δ_), it was found that after adding 50 ppm H_2_S to hydrogen, the power densities decreased over the first 46 h due to sulfur poisoning and the formation of a needle-like iron sulfide structure. However, over the next 300 h of operations, power density stabilized, which may be associated with the established equilibrium between the formation and removal of sulfides in the form of sulfur oxide SO_2_. Similar results were obtained in [21]: when testing a symmetrical SOFC (Sr_2_Fe_1.5_Mo_0.5_O_6-δ_|LSGM|Sr_2_Fe_1.5_Mo_0.5_O_6-δ_, SFM|LSGM|SFM), its power characteristics decreased by 10% after replacing pure H_2_ with 100 ppm H_2_S; however, after electrode regeneration in air at 800 °C, the power densities reached their initial values (Figure 16).

With the direct supply of methane to the same symmetric cell, power density deterioration was also registered. However, the power density stabilized after annealing in air at 800 °C (Figure 16). This effect was attributed to the formation of carbon by Reaction (11), which subsequently burns in oxygen at high temperatures. In conclusion, the authors noted that Sr_2_Fe_1.5_Mo_0.5_O_6-δ_ is a highly promising candidate.

## 6. Conclusions

According to the literature overview, double perovskites (Sr_2_MMoO_6-δ_, where M = Ni, Mg, Fe) are promising materials for use as fuel electrodes in SOFCs. More precisely, some of them demonstrate a high tolerance for sulfur poisoning and carbonization. However, the optimal compositions have not yet been identified for undoped double molybdates, which is due to the difficulty of achieving the required set of target properties. For example, some complex oxides are stable in oxidizing conditions (which are realized during the joint sintering of SOFCs), but decompose in reducing atmospheres (in which anodes must operate effectively). Others, in contrast, exhibit a single phase form in a reduced state, but become a multiphase system when in oxidizing conditions, which, as a rule, leads to the degradation of many functional properties. It is evident that optimization strategies rest on the further modification of Sr_2_MMoO_6-δ_ using single or multi-doping (see examples in Table 8). Although the effects of such doping were not considered within the present review, this works gives basic data on the natural properties of Sr_2_MMoO_6-δ_, acting a starting point for designing modernized double perovskite molybdate derivatives for energy conversion and electrochemical purposes.

## Figures and Tables

**Table 1:** Performance of SOFCs based on double Sr_2_MMoO_6-δ_ molybdates and La_1-x_Sr_x_Ga_1–y_Mg_y_O_3-δ_ (LSGM) electrolytes.

Electrolyte Thickness, µm	Cathode Composition	Conditions	P_max_, mW cm^−2^	Ref.
Sr_2_NiMoO_6-δ_				
300	SrCo_0.8_Fe_0.2_O_3-δ_	H_2_, 800 °C 3% H_2_O/CH_4_, 800 °C CH_4_, 800 °C	480 110 270	[24]
300	Ba_0.5_Sr_0.5_Co_0.8_Fe_0.2_O_3-δ_	H_2_, 850 °C H_2_, 800 °C H_2_, 750 °C	820 595 400	[75]
Sr_2_MgMoO_6-δ_				
280	Ba_0.5_Sr_0.5_Co_0.8_Fe_0.2_O_3-δ_	H_2_, 800 °C	660	[76]
300	SrCo_0.8_Fe_0.2_O_3-δ_	H_2_, 800 °C CH_4_, 800 °C	840 440	[77]
300	Ba_0.5_Sr_0.5_Co_0.8_Fe_0.2_O_3-δ_	H_2_, 850 °C H_2_, 800 °C CH_4_, 850 °C CH_4_, 800 °C	860 600 605 430	[40]
300	SmBaCo_2_O_5+δ_	H_2_, 850 °C H_2_, 800 °C H_2_, 750 °C	830 585 410	[78]
300	Ba_0.5_Sr_0.5_Co_0.8_Fe_0.2_O_3-δ_	H_2_, 800 °C	520	[79]
1200	Sr_2_Fe_1.5_Mo_0.5_O_6-δ_	3% Н_2_О/Н_2_, 800 °C	375	[80]
30	La_0.8_Sr_0.2_MnO_3_–YSZ	H_2_, 850 °C	667	[81]
30	La_0.8_Sr_0.2_MnO_3_–YSZ	biogas, 850 °C	520	[81]
400	Sr_2_MnMoO_6-δ_/NiO–Ce_0.8_Sm_0.2_O_2-δ_	CH_4_, 800 °C	245	[28]

**Table 2:** Thermal expansion coefficients of Sr_2_NiMoO_6-δ_ in air. These data are also presented in Figure A1.

Temperature Range, °C	α·10^6^, K^−1^	Ref.
27–950	12.1	[75]
30–575	12.4	[92]
575–1100	14.0	[92]
20–1300	12.9	[93]

**Table 3:** Conductivity of Sr_2_NiMoO_6-δ_. These data are also presented in Figure A2.

Measuring Conditions	σ, S cm^−1^	Ref.
5%H_2_/Ar, 800 °C	0.1	[24]
H_2_, 800 °C	1.1	[24]
CH_4_, 800 °C	1.1	[24]
H_2_, 800 °C	1.6	[39]
H_2_, 850 °C	49	[75]
pO_2_ = 1 × 10^−6^ Pa, 600 °C	7·10^−4^	[88]

**Table 4:** Thermal expansion coefficients of Sr_2_MgMoO_6-δ_ in air. These data are also presented in Figure A1.

Temperature Range, °C	α·10^6^, K^−1^	Ref.
109–360	11.7	[77]
360–800	12.7	[77]
25–800	15.1	[100]
50–1300	12.9	[104]
n/a	13.6	[113]

**Table 5:** Conductivity of Sr_2_MgMoO_6-δ_. These data are also presented in Figure A2.

Measuring Conditions	σ, S cm^−1^	Ref.
pO_2_ = 10^−24^, 800 °C	3.5	[18]
5%H_2_/Ar, 800 °C	9.5	[45]
5%H_2_/Ar, 800 °C	4	[77]
H_2_, 800 °C	10	[77]
5%H_2_/Ar, 800 °C	0.07	[97]
5%H_2_/N_2_, 800 °C	50	[100]
5%H_2_/Ar, 900 °C	0.5	[104]
5%H_2_/Ar, 800 °C	1.5	[113]

**Table 6:** Conductivity of Sr_2_Fe_1.5_Mo_0.5_O_6–__δ_. These data are also presented in Figure A2.

Measuring Conditions	σ, S cm^−1^	Ref.
H_2_, 800 °C	9	[78]
Air, 800°C	13	[80]
Air, 800°C	10	[98]
H_2_, 800 °C	16	[125]
Air, 800°C	17	[126]
H_2_ (3 % H_2_O)_,_ 800°C	13	[127]
H_2_, 780 °C	310	[135]
Air, 780 °C	550	[135]
H_2_, 800 °C	41	[136]

**Table 7:** Thermal expansion coefficients of Sr_2_Fe_1.5_Mo_0.5_O_6-__δ_ in air. These data are also presented in Figure A1.

Temperature Range, °C	α·10^6^, K^−1^	Ref.
200–760	14.5	[122]
760–1200	21.4	[122]
200–1200	18.1	[122]
40–350	11.6	[123]
500–800	18.6	[123]
40–950	16.3	[124]
50–450	12.8	[126]
650–900	20.2	[126]

**Table 8:** Doping strategies performed for tailoring the functional properties of Sr_2_MMoO_6-δ_.

System	Concentration, x	Ref.
Sr_2_NiMoO_6-δ_		
Sr_2-x_Ce_x_NiMoO_6-δ_	x = 0.01	[139]
Sr_2-x_Ce_x_NiMoO_6-δ_	0 ≤ x ≤ 0.05	[140]
Sr_2-x_Sm_x_NiMoO_6-δ_	0 ≤ x ≤ 0.05	[90]
Sr_2-x_La_x_NiMoO_6-δ_	0 ≤ x ≤ 0.1	[141]
Sr_2-x_Ba_x_NiMoO_6-δ_	0 ≤ x ≤ 1	[142]
Sr_2_Ni_1-x_Mg_x_MoO_6-δ_	0 ≤ x ≤ 0.25	[143,144,145,146,147]
Sr_2_Ni_1-x_Mg_x_MoO_6-δ_	x = 0.3	[148]
Sr_2_Ni_1-x_Mg_x_MoO_6-δ_	0 ≤ x ≤ 0.75	[149,150,151]
Sr_2_Ni_1-x_Mg_x_MoO_6-δ_	0 ≤ x ≤ 1	[25]
Sr_2_Ni_1-x_Zn_x_MoO_6-δ_	0 ≤ x ≤ 1	[142]
Sr_2_MgMoO_6-δ_		
Sr_2_MgMo_1-x_Co_x_O_6-δ_	x = 0.1	[110,152,153]
Sr_2_MgMo_1-x_Mn_x_O_6-δ_	x = 0.1	[110,152,153]
Sr_2_MgMo_1-x_Ni_x_O_6-δ_	x = 0.1	[110,152,153,154]
Sr_2-x_Ca_x_MgMoO_6-δ_	0 ≤ x ≤ 0.5	[113]
Sr_2_FeMoO_6-δ_		
Sr_2_FeMo_1-x_Mg_x_O_6-δ_	x = 1/3	[155]
Sr_2_FeMo_1-x_Nb_x_O_6-δ_	0 ≤ x ≤ 1	[156]
Sr_2-x_Nd_x_FeMoO_6-δ_	0 ≤ x ≤ 0.05	[157]
Sr_2-x_La_x_FeMoO_6-δ_	0 ≤ x ≤ 1	[120,158]
Sr_2-x_Ba_x_FeMoO_6-δ_	0 ≤ x ≤ 2	[120,159]
Sr_2_Fe_1.5_Mo_0.05_O_6-δ_		
Sr_2_Fe_1.5-x_Cu_x_Mo_0.5_O_6-δ_	0 ≤ x ≤ 0.3	[160]
Sr_2_Fe_1.5-x_Ni_x_Mo_0.5_O_6-δ_	0 ≤ x ≤ 0.4	[123,161,162]
Sr_2_Fe_1.5-x_Ga_x_Mo_0.5_O_6-δ_	x = 0.2	[163]
Sr_2_Fe_1.5-x_Co_x_Mo_0.5_O_6-δ_	x = 0.2	[164]
Sr_2_Fe_1.5-x_Co_x_Mo_0.5_O_6-δ_	0 ≤ x ≤ 1.0	[126]
Sr_2_Fe_1.5-x_Nb_x_Mo_0.5_O_6-δ_	x = 0.1	[80]
Sr_2_Fe_1.5-x_Mn_x_Mo_0.5_O_6-δ_	x = 0.1	[165]
Sr_2_Fe_1.4-x_Ti_x_Mo_0.6_O_6-δ_	0 ≤ x ≤ 0.1	[166]
Sr_2_Fe_1.5_Mo_0.5-x_Zr_x_O_6-δ_	x = 0.1	[167]
Sr_2_Fe_1.5–3x_Mo_0.5-x_Co_4x_O_6-δ_	x = 0.05	[168]
Sr_2-x_La_x_Fe_1.5_Mo_0.5_O_6-δ_	x = 0.5	[169,170]
Sr_2-x_Ca_x_Fe_1.5_Mo_0.5_O_6-δ_	0 ≤ x ≤ 0.6	[171]
Sr_2_Fe_1.5_Mo_0.5_O_6-δ-x_Cl_x_	0 ≤ x ≤ 0.4	[172]
Sr_2_Fe_1.5_Mo_0.5_O_6-δ-x_F_x_	0 ≤ x ≤ 0.3	[173]

**Figure A1:**
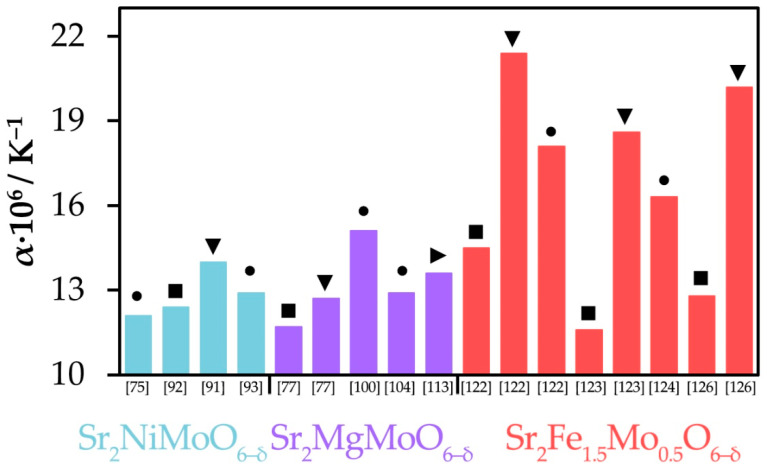
Thermal expansion coefficients (α) of Sr_2_MMoO_6-δ_ materials in air. Visualization of Table 2, Table 4 and Table 7. The α values were calculated within entire- (●), low- (■) and high- (▼) temperature ranges. The ► marker indicates that the data are not applicable.

**Figure A2:**
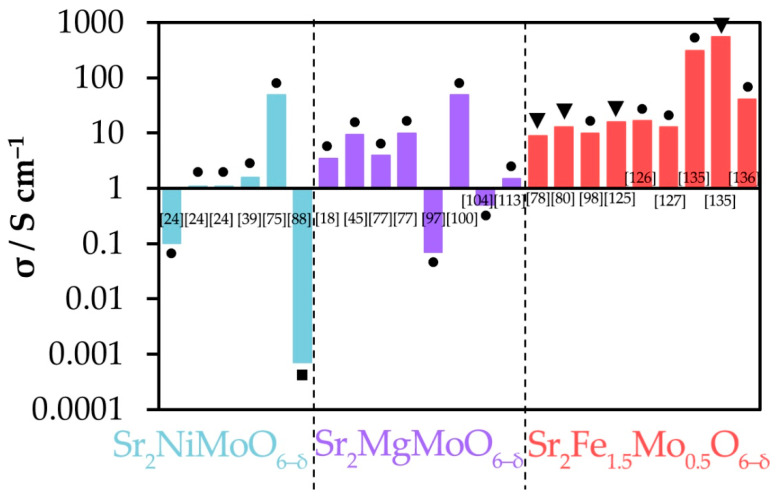
Total conductivity of Sr_2_MMoO_6-δ_ materials in reducing (●), pO_2_-intermediate (■) and oxidizing (▼) atmospheres. Visualization of Table 3, Table 5 and Table 6.

## Data Availability

Data sharing not applicable.
